# Teaching old dogs new tricks: genetic engineering methanogens

**DOI:** 10.1128/aem.02247-23

**Published:** 2024-06-10

**Authors:** Tyler Myers, Christy M. Dykstra

**Affiliations:** 1 Department of Civil, Construction and Environmental Engineering, San Diego State University, San Diego, California, USA; 2 Department of Bioengineering, University of California San Diego, La Jolla, California, USA; Kyoto University, Kyoto, Japan

**Keywords:** archaea, CRISPR, Euryarchaeota, genetic tools, mutagenesis, synthetic biology, transformation

## Abstract

Methanogenic archaea, which are integral to global carbon and nitrogen cycling, currently face challenges in genetic manipulation due to unique physiology and limited genetic tools. This review provides a survey of current and past developments in the genetic engineering of methanogens, including selection and counterselection markers, reporter systems, shuttle vectors, mutagenesis methods, markerless genetic exchange, and gene expression control. This review discusses genetic tools and emphasizes challenges tied to tool scarcity for specific methanogenic species. Mutagenesis techniques for methanogens, including physicochemical, transposon-mediated, liposome-mediated mutagenesis, and natural transformation, are outlined, along with achievements and challenges. Markerless genetic exchange strategies, such as homologous recombination and CRISPR/Cas-mediated genome editing, are also detailed. Finally, the review concludes by examining the control of gene expression in methanogens. The information presented underscores the urgent need for refined genetic tools in archaeal research. Despite historical challenges, recent advancements, notably CRISPR-based systems, hold promise for overcoming obstacles, with implications for global health, agriculture, climate change, and environmental engineering. This comprehensive review aims to bridge existing gaps in the literature, guiding future research in the expanding field of archaeal genetic engineering.

## INTRODUCTION

The discovery of methane- (CH_4_-) producing microbes with DNA sequences distinct from bacterial DNA led to the establishment of Archaea as a new domain of life (1). Methanogens, which exhibit unique physiological and biochemical features, contribute to global carbon and nitrogen cycling, and inhabit diverse environments, including soils, ocean sediments, and the human/animal microbiome (2, 3). Most known methanogens belong to the phylum Euryarchaeota, which consists of a wide range of diverse archaeal classes that may be grouped by their distinct physiological or metabolic characteristics (1). According to the taxonomy proposed by Cavalier-Smith and Chao (2), Euryarchaeota consists of the classes: *Thermococcia*, *Thermoplasmia*, *Methanobacteriia*, *Methanocellia*, *Archaeoglobia*, and *Halobacteria*. However, debate remains around the proper taxonomic classification of groups including *Methanomicrobia*, *Methanopyri*, and *Methanococci* (1). Regardless of taxonomic placement, known methanogens belong to the following classifications: *Thermoplasmia*, *Methanobacteriia*, *Methanocellia*, *Methanonatronarchaeia*, *Methanomicrobia*, *Metanephric*, and *Methanococci* (1–4).

The development of new archaeal genetic tools can help improve our understanding of the role of methanogens in both natural and engineered environments. To date, most genetic engineering tools have been developed for well-studied model organisms within the Bacteria or Eukaryote domains, such as *Escherichia coli* and *Saccharomyces cerevisiae* (5, 6). Yet, many of these genetic tools are unsuitable for methanogens because of physiological differences and the relatively understudied nature of archaeal genetic systems. Furthermore, some methanogens have slow growth rates and/or require difficult cultivation conditions (e.g., extreme pressure, salinity, pH, temperature, and/or co-cultivation) (7). Methanogens also have a strictly anaerobic metabolism, which adds complexity to species isolation and genetic manipulation (8). The archaeal transformation screening process can be more difficult than bacterial transformation screening because many archaea are resistant to conventional bacterial antibiotics (i.e., β-lactams, tetracyclines, and phenicols), or must be cultivated in conditions that are detrimental to bacterial selection markers (9, 10). However, recently developed genetic tools, including CRISPR-based systems, promise to tackle these challenges and expand the use of archaeal-based genetic engineering for many different applications, including global health, agriculture, climate change, and the environment (11). Because of the fast pace of recent archaeal genetic tool development, current literature lacks an up-to-date, comprehensive review of genetic engineering tools for methanogens. Therefore, the objective of this review is to describe pivotal research and the state-of-the-art of genetic engineering in methanogens.

## HOST METHANOGENS

Archaeal and bacterial cell membranes exhibit fundamental differences that pose challenges, or even prevent, the application of certain bacterial genetic engineering methods to methanogens. Thus, methods for cell membrane disruption to insert genetic material, and methods for selection and counterselection (see “Selection and counterselection markers”), differ significantly between Archaea and Bacteria. The structure and permeability of a cell envelope directly influence a methanogen’s resistance to extreme environments (12, 13). During physicochemical mutagenesis, the cell must be treated to allow for genetic material to enter the cell, while not damaging the cell as to negatively affect growth and/or viability. The diversity of cell wall construction among methanogens (Table 1) means that a “one-size fits all” approach to cell disruption and genetic manipulation is not appropriate, even between different methanogens (12).

**TABLE 1:** Cell wall construction and morphology of select methanogens

Order	Species	Cell envelope^*a*^	Morphology	Reference
*Methanopyri*	*Methanopyrus kandleri*	Pseudomurein	Rods	(12)
*Methanobacteriales*	*Methanothermus fervidus*	S-layer, Pseudomurein	Rods	(12)
	*Methanothermobacter thermoautotrophicus*	Pseudomurein	Rods	(12)
	*Methanobrevibacter ruminantium*	Pseudomurein	Rods	(12)
	*Methanobrevibacter fomicum*	Pseudomurein	Rods	(12)
*Methanococcales*	*Methanococcus jannaschii*	S-layer	Irregular cocci	(14)
	*Methanococcus maripaludis*	S-layer	Pleomorphic coccoid-rod	(15)
	*Methanococcus vannielii*	S-layer	Irregular cocci	(16)
	*Methanococcus voltae*	S-layer	Irregular cocci	(12)
*Methanosarcinales*	*Methanosarcina mazei*	Methanochondroitin, S-layer	Single cells, Aggregates, Pseudosarcina	
	*Methanosarcina acetivorans*	Methanochondroitin, S-layer	Irregular cocci, Single cells, Aggregates	(17)
	*Methanolobus tindarius*	S-layer	Cocci	(18)
	*Methanosaeta concilii*	Proteinaceaous sheath and analog to S-layer	Sheathed rods	(12)
*Methanomicrobiales*	*Methanospirillum hungatei*	Proteinaceaous sheath, S-layer	Curved rods	(19)
	*Methanocorpusculum parvum*	S-layer	Irregular cocci	(20)
	*Methanocorpusculum sinense*	S-layer	Irregular cocci	(21)
	*Methanocorpusculum bavaricum*	S-layer	Irregular cocci	(21)
	*Methanoculleus marisnigri*	S-layer	Irregular cocci	(22)
	*Methanolacinia paynteri*	S-layer	Pleomorphic coccoid-rod	(21)
	*Methanoplanus limicola*	S-layer	Flat plate	(23)
	*Methanogenium cariaci*	S-layer	Irregular cocci	(24)
	*Methanogenium tationis*	S-layer	Irregular cocci	(24)
	*Methanogenium marisnigri*	S-layer	Irregular cocci	(24)

^*a*^ Cell envelope composition from Albers and Meyer (25).

The archaeal cell membrane is made of isoprenoid alkyl chains linked by ether bonds to glycerol-1-phosphate. In contrast, Bacteria have a cell membrane made of fatty acids linked to glycerol-3-phosphate by ester bonds (25). The more stable ether bond in the archaeal membrane supports survival in extreme conditions that may be inhospitable to many Bacteria, such as high temperatures, high/low pH, high salinity, and high degrees of mechanical stress (25–27). Although these characteristics make methanogens desirable for many biotechnology applications, they also make the cell envelope difficult to disrupt in a controlled manner for genetic manipulation. Some methanogens have a cell wall that consists of pseudomurein, a polymer that is structurally similar to murein in bacterial cell walls. Other methanogens have a surface layer (S-layer) crystalline protein network built of multiple copies of one to two glycosylated proteins instead of, or in addition to, pseudomurein. For example, *Methanobacteriia* are a class of methanogens with cell walls usually made of pseudomurein, although a few known species have an S-layer (2). Murein and pseudomurein are similar in structure but have different bonds that form between sugars. In murein, a β-1,4-glycosidic bond connects N-acetylglucosamine and N-acetylmuramic acid; in pseudomurein, a β-1,3-glycosidic bond connects N-acetylglucosamine to N-acetyltalosaminuronic acid or N-acetylgalactosamine (28). Lysozyme that is used for bacterial cell disruption (e.g., DNA/RNA extraction) is not effective in methanogens because the sugar linkage makes pseudomurein resistant to lysis. Thus, methanogens require specialized pseudomurein-degrading enzymes, such as the pseudomurein endoisopeptidases PeiW and PeiP, which are encoded by the *Methanothermobacter wolfeii* prophage PsiM100 and the *Methanothermobacter marburgensis* phage PsiM2, respectively (29). One method to lyse *Methanothermobacter thermautotrophicus* involves alkaline lysis and incubation with PeiP at high temperatures (30).

Host methanogen growth conditions (e.g., temperature, salinity, etc.) may also influence the cell membrane structure and permeability (12, 13). For example, some methanogens, such as *Methanosarcina* spp., natively form cell aggregates that are “glued” together with methanochondroitin, which is an exopolysaccharide that interacts with the S-layer around the cell envelope. This aggregation of cells presents a physical barrier to genetic transfer; however, under highly saline conditions, the production of methanochondroitin is suppressed and cells may grow without aggregation (31).

In many cases, the selection of a methanogenic host to use for genetic engineering will be determined by the specific research goal. However, some research supporting biotechnology applications may not require a specific type of methanogen but, rather, a specific outcome (e.g., improved CH_4_ production). In these cases, and in other instances where the research goal is not highly specific for one particular methanogen, the selection of a proper methanogenic host is the first step in the genetic engineering process. A recent review by Costa and Whitman (32) provides a comprehensive overview of current model methanogen species. The present review instead aims to focus on describing current genetic tools for methanogens.

## GENETIC TOOLS

**Fig 1:**
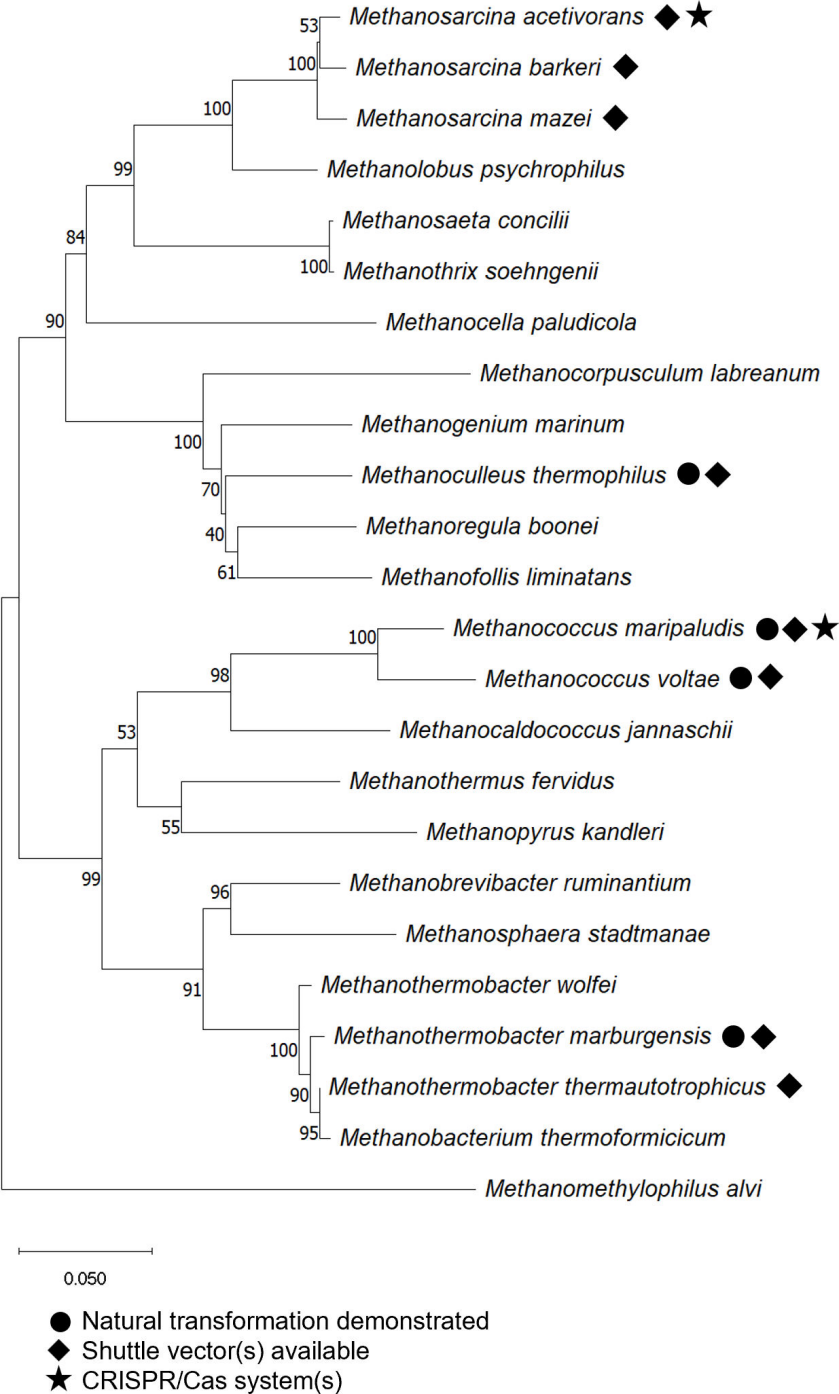
Phylogenetic tree of methanogens and developed genetic systems and tools. GenBank 16S rRNA gene sequences were aligned with ClustalW and the tree was constructed using the neighbor-joining method in Mega11.

Recently developed genetic tools (Fig. 1) have revolutionized the understanding of methanogens and their place within the evolutionary tree of life (1, 2). As genetic tools continue to advance, greater insights into the biology and unique adaptations of methanogens can be acquired, and biotechnology applications may be advanced in areas including wastewater energy recovery, carbon recycling, and sustainable chemical production.

### Selection and counterselection markers

To select cells that were successfully transformed, an antibiotic resistance gene is often included in the transferred genetic material. For example, the *pac* gene, which encodes for the enzyme puromycin N-acetyl-transferase and confers resistance to the antibiotic puromycin, was used for selection in the first reported transformations of *Methanococcus voltae* and *M. maripaludis* (33, 34). In contrast, counterselection selects for cells that have not been modified, and is frequently used to identify or isolate cells that have reverted to a wild-type state or have lost a particular genetic element. For example, the purine analog 8-aza-2,6-diaminopurine (8ADP) may be used as a counterselection agent for methanogens that develop resistance to 8ADP when the *hpt* gene, which encodes for hygromycin phosphotransferase, is inactivated through mutation (35).

As discussed in “Host methanogens,” the cell envelope of methanogenic Archaea differs significantly from Bacteria, which also results in different susceptibility to antibiotics used as selection markers. Antibiotics that inhibit murein synthesis, such as penicillin and vancomycin, are not effective in methanogens because archaeal cells contain pseudomurein instead of murein, and the synthesis pathways differ (12). Another antibiotic, tetracycline, works by binding to the bacterial ribosome, which is responsible for protein synthesis. In contrast, Archaea have a different ribosomal structure and protein synthesis machinery and, therefore, are not susceptible to tetracycline antibiotics (9). Moreover, some methanogens even have resistance to antibiotics and counterselection markers that are frequently used with other methanogens, such as puromycin, neomycin, and 8ADP. The hyperthermophile *Methanocaldococcus jannaschii* is not inhibited by typical antibiotic concentrations of neomycin, puromycin, or novobiocin, and is resistant to several base analogs that are often used for counterselection, such as 6-methylpurine, 6-thioguanine, 6-azauracil, 5-fluorouracil, 8-azahypoxanthine, and 8ADP (36).

*Methanosarcina acetivorans* and *Methanosarcina barkeri* are susceptible to puromycin, 8ADP, and pseudomonic acid, but they are not known to be susceptible to neomycin or the related antibiotic G-418 (37–39). However, the related *Methanosarcina mazei* is susceptible to neomycin and, in one study, a neomycin resistance selection marker was developed (40). *Methanococcus* species tend to be susceptible to puromycin and neomycin, although specific strains may have unique resistance to antibiotics (41, 42). For example, the *M. maripaludis* S0001 strain is resistant to 8‐azahypoxanthine and 6‐azauracil because of gene deletions that encode for hypoxanthine and uracil phosphoribosyltransferase, respectively (43). In addition, antibiotic concentrations are important because cells may alter the copy number of antibiotic resistance plasmids in response to high antibiotic concentrations, thereby increasing resistance levels (44).

### Reporter systems

Reporter systems are used to identify successful transformation, study gene expression, and measure cellular processes. For example, the *uidA* gene, which encodes the enzyme β-glucuronidase (GUS), may be used for colorimetric assays. When the *uidA* gene is expressed, GUS hydrolyzes 5-bromo-4-chloro-3-indolyl glucuronide (X-Gluc) into glucuronic acid and produces the blue-colored product, 5,5′-dibromo-4,4′-dichloro-indigo (38). Thus, insertion of *uidA* alongside a target gene has been used for colorimetric assay for gene expression (38, 45).

Similarly, the *lacZ* gene, which encodes β-galactosidase, has also been used as a reporter in several genetic studies of methanogens. When *lacZ* is expressed, colorless X-gal (5-bromo-4-chloro-3-indolyl-beta-d-galactopyranoside) is converted into a blue product for colorimetric assay. Notably, *lacZ* has been used as a reporter gene, together with directed mutagenesis, to identify a repressor binding site that regulates transcription of the *nifH* gene in *M. maripaludis* (46). *lacZ* has also been used as a reporter to study formate dehydrogenase gene clusters in *M. maripaludis* (47), aspartate aminotransferase in *Methanobacterium thermoformicicum* strain SF-4 (48), and the *cofD* gene in *Methanobrevibacter ruminantium* (49).

Although methanogens lack susceptibility to β-lactam antibiotics, a β-lactamase reporter system that used the chromogenic substrate nitrocefin under anaerobic conditions was developed for *M. acetivorans* (50). This study examined the effect of the transcriptional regulator HrsM on the selenium-dependent expression of the hydrogenase gene *frcA* in *M. maripaludis* (51). A β-lactamase reporter system was also used to probe the translation of selenocysteine (Se) in *M. maripaludis*. Se is an unusual amino acid that is encoded by translational re-coding, instead of direct gene coding. Thus, the UGA codon, which normally terminates translation, instead codes for Se in the presence of a selenocysteine insertion sequence. Interestingly, methanogens are the only Archaea that have been experimentally verified to have Se (52). These studies highlight the possible use of β-lactamase reporter systems in methanogens, despite the lack of methanogen susceptibility to β-lactam antibiotics.

Common fluorescence proteins, such as Green Fluorescence Protein (GFP) and mCherry, require oxygen to fluoresce, which limits their use with obligately anaerobic methanogens. Instead, these fluorescent reporters can be used to report expression in non-native microorganisms (e.g., *E. coli*). For example, GFP was used to characterize the pseudomurein cell wall-binding domain in *M. thermautotrophicus* via expression in *E. coli* (53). A dual-fluorescent protein reporter system with Blue Fluorescence Protein (BFP) and GFP was used to demonstrate the incorporation of noncanonical amino acids into *S. cerevisiae* using pyrrolysyl-tRNA synthetase from *Methanomethylophilus alvus* (54). Although GFP and mCherry can be directly expressed in methanogens, fluorescence will not occur without lethal air exposure over several hours to allow for chromophore maturation (55). Despite the destructive nature of this method, mCherry has been used in *M. maripaludis* to evaluate the function of the *Methanolobus psychrophilus* R15 *mtaCB* operon (56).

More recently, fluorescence-activating and absorption-shifting tags (FAST and FAST2) have been developed that function under anaerobic conditions and have been applied to methanogens, which expands the available molecular tools to include fluorescent-based protein localization, high-throughput flow cytometry, and fluorescence-activated cell sorting (57–59). FAST and FAST2 are fluorogen-activating proteins that cause small fluorogens to fluoresce brightly upon binding with the protein. This type of reporter system only requires a small monomeric protein (e.g, 14 kDa, 125 amino acid residues), and can be used with several fluorogens that have different absorption and emission wavelengths (60, 61). For example, a FAST2 tag was used in *M. acetivorans* and *M. maripaludis* to conduct flow cytometry, which may then be used to quantify target methanogens in multi-population systems (57). Thus, FAST reporters are a promising tool for accelerating methanogen gene and protein studies.

### Shuttle vectors

Shuttle vectors are genetic elements (i.e., DNA or plasmids) that are capable of replication and maintenance in multiple hosts by using multiple origins of replication, with each replication sequence specific to one host. *E. coli* is the most common partner host for shuttle vectors in methanogens because of its versatile and well-developed genetic techniques (62). Replicating vectors can also be useful for introducing genetic constructs without any residual host genome modifications, as discussed further in “Markerless genetic exchange.”

A number of shuttle vectors have been developed for methanogens (Table 2). Today, the most extensive methanogen shuttle vector toolkit has been developed for *M. maripaludis*, and most of the vectors utilize puromycin resistance as a marker (63–68). Shuttle vectors with neomycin resistance have also been extensively used in *M. maripaludis* (42, 52, 69–73). Additionally, a shuttle vector for *M. maripaludis* with 6-azauracil resistance has been established (70).

**TABLE 2:** Methanogens and their associated shuttle vector plasmids, selection, and counterselection markers

Methanogen	Strain(s)	Shuttle vector(s)	Selection/counterselection					References
			Pur^*a*^	Neo^*b*^	8ADP^*c*^	PA^*d*^	Other	
*Methanococcus*								
*M. maripaludis*	Multiple	pWLG40NZ–R	X^*e*^	X				(42)
	Multiple	pMEV2	X	X				(69)
	Multiple	pKAS100, pKAS102	X					(64)
	Multiple	pMEV4	X					(68)
	Multiple	pMEV4	X					(74)
	JJ	pLW40, pLW40neo	X	X			6A^*f*^	(70)
	JJ	pLW40neo		X				(71)
	JJ	pWLG13, pWLG30	X					(63)
	JJ	pWLG40NZ-R		X				(52)
	JJ	pKAS102	X					(67)
	JJ	pDLT44	X					(66)
	JJΔupt	pMM002P, pMM005	X					(75)
	S0001	pMEV5mT-P243	X					(65)
	S0001	pMEV2-0076, pIN-0076	X	X				(73)
	ΔmmpX	pMEV5mT-P243	X					(65)
	S2	pLW40, pLW40neo	X	X			6A	(70)
	S2	pEFJ9		X				(72)
	S2	pAW42	X					(76)
*M. voltae*	PS	pUC						(77)
	PS	pKAS102, pJLA5, pJLA6	X	X				(78)
	DSM 1537	pINT, pIR	X					(79)
*Methanosarcina*								
*Methanosarcina* spp.	Multiple	pWM307	X					(41)
*M. acetivorans*	Multiple	pDL730	X					(80)
	C2A	pJK89	X		X			(38)
	C2A	pPB35	X			X		(39)
	WWM73	pMCp4	X		X			(81)
*M. barkeri*	Fusaro	pWM307	X			X		(37)
*M. mazei*	N/A ^*g*^	pRS1595	X					(62)
	Gӧ1	pRS283	X		X			(35)
	Gӧ1	pWM321	X	X				(40)
*Methanothermobacter*								
*M. thermautotrophicus*	ΔH	pMVS		X				(30)
	ΔH	pMVS1111A		X				(44)
*M. marburgensis*	N/A	pET2411						(82)
*Methanoculleus*								
*M. thermophilus*	DSM 2373	pJAL1inter		X			6A, 8AH^*h*^	(70)

^*a*^ Pur, puromycin.

^*b*^ Neo, neomycin.

^*c*^ 8ADP, 8-aza-2,6-diaminopurine.

^*d*^ PA, pseudomonic acid.

^*e*^ X indicates the selection/counterselection markers applicable to the methanogen.

^*f*^ 6A, 6-azauracil.

^*g*^ N/A, not available.

^*h*^ 8AH, 8-azahypoxanthine.

The first discovered plasmid for a methanogen, pME2001, was joined with other plasmids to develop potential shuttle vectors between *M. marburgensis* (previously classified as *Methanobacterium thermoautotrophicum*) and other common hosts, including *E. coli*, yeast, *Bacillus subtilis*, and *Staphylococcus aureus*. One promising vector, pET2411, could replicate in *E. coli* in the presence of *E. coli* DNA polymerase I; however, the vector only carried genes for selection in *E. coli* and contained no marker for methanogen selection (82). Later, a shuttle vector with a marker for neomycin resistance was developed for the related species, *M. thermautotrophicus* ΔH (30), and used in subsequent gene expression studies (44, 83).

A shuttle vector (PWM307) that would replicate in multiple *Methanosarcina* species was used to develop a selection marker for pseudomonic acid resistance in *M. barkeri* Fusaro (37, 41). Multiple shuttle vectors have since been developed for *M. acetivorans* using puromycin resistance as a selection marker (38, 39, 80, 81). The shuttle vectors, pJK89 and pMCp4, include 8ADP selection (38, 81), and one vector, pPB35, includes pseudomonic acid selection (39). A shuttle vector with a puromycin selection marker was also demonstrated in *M. mazei* (40). A shuttle vector that also conferred 8ADP resistance (35), and a vector with a neomycin resistance marker (40), were developed. Within *Methanoculleus thermophilus*, a shuttle vector with neomycin and additional markers was constructed (70).

The BioBrick synthetic biology approach involves standardizing DNA fragments that encode various biological components, assembling these standardized parts to create new biological devices, and enabling global reusability of BioBrick parts for building complex biological systems. BioBrick parts are standardized genetic elements and have been instrumental in advancing the development of genetically engineered organisms, metabolic engineering, and synthetic biology. Although relatively few BioBrick-compatible parts have been developed for methanogens to date, some BioBrick-compatible shuttle vectors are available for *M. maripaludis* (84–86). Another BioBrick-compatible shuttle vector was developed in a study that evaluated the effect of high temperature and osmolarity conditions on *Methanothermus fervidus* (87). Further development of BioBrick and other standard genetic elements for methanogens will accelerate the pace of genetic discovery and innovation.

## MUTAGENESIS

Random mutagenesis of methanogens may be achieved using physicochemical processes (i.e., chemical mutagenesis, UV/gamma irradiation, etc.) and transposon-based mutagenesis; in contrast, site-directed mutagenesis may be achieved with liposome-mediated mutagenesis and markerless genetic exchange methods (see “Markerless genetic exchange”). Random mutagenesis may be used to explore the genome of a methanogen in which gene functions are unknown, and is most useful for interrogating lesser-studied methanogens for which gene annotation is lacking. In contrast, site-directed mutagenesis provides a higher level of control and specificity of the genetic manipulation, and is suited for research into specific genetic changes, or studying the structure-function relationships of proteins.

### Natural transformation

Natural transformation is thought to occur less frequently in Archaea than in Bacteria (88). However, some competent methanogens that more readily undergo natural transformation have been discovered and may be used for simplified gene manipulation. Natural transformation in a methanogen was first demonstrated with *M. marburgensis* and *M. voltae* (34, 89). Later, puromycin resistance was conferred to *M. voltae* PS, a strain with a relatively high transformation frequency (705 transformants per µg of transforming DNA) compared to other *M. voltae* strains (i.e., 9 transformants µg^−1^ DNA) (67, 90). More recently, highly competent strains of *M. maripaludis* (2.4 × 10^3^ transformants µg^−1^ DNA) and *M. thermophilus* (2.7 × 10^3^ transformants µg^−1^ DNA) were identified (70). These naturally competent strains reduce the need for complex transformation methods and speed up the pace of genetic manipulation.

Mechanism(s) of archaeal natural transformation are not currently well-defined. In many competent Bacteria, double-stranded DNA (dsDNA) first binds to a type IV pilus or a transformation-specific pilus. A nuclease then converts the dsDNA to single-stranded DNA (ssDNA) before the DNA enters the cytoplasm via Com transporter proteins (88, 91). Although a similar mechanism may exist in Archaea, archaeal homologs have not yet been identified for one of the Com proteins, ComEC (88). In competent strains of *M. maripaludis* and *M. thermophilus*, type IV pilus is required for DNA uptake, and that competence may be conferred to a noncompetent *M. maripaludis* strain by expressing the pilin genes (70). A study using random mutagenesis identified genes necessary for natural transformation, which included genes encoding for type IV pilus, membrane-bound transporters, and other unknown genes (71).

Non-competent methanogens may require specific environmental conditions, such as quorum sensing cues, low substrate concentrations, or other stressors, to induce competence. For example, competence in *M. maripaludis* S2 must be induced by expressing pilin proteins, although *M. maripaludis* strain JJ is constitutively competent (71). The study of archaeal natural transformation is still a developing field; thus, new genetic tools may yield additional insights.

### Physicochemical mutagenesis

Early physicochemical mutagenesis of a methanogen used UV irradiation or gamma rays to induce mutations in *Methanococcus voltae* (34). Electroporation was then used to transform protoplasts of *M. voltae*, which achieved a 380-fold increased transformation efficiency over natural transformation (90). A polyethylene glycol (PEG)-mediated transformation method with a pKAS102 integration vector was also developed to transform *M. maripaludis*, which achieved a four-fold higher rate of transformation than under natural transformation (67). PEG-mediated transformation induces a protoplast state in the cell, which allows for the uptake of linear or circular (i.e., plasmid) DNA (88). However, other more efficient methods of random mutagenesis have since been developed, as described next (see “Transposon-mediated mutagenesis”).

### Transposon-mediated mutagenesis

*In vitro* transposon mutagenesis was first demonstrated in *M. maripaludis* while studying the role of the *nifH* gene. The target DNA was cloned into *E. coli* and the transposon-induced mutagenesis occurred within the *E. coli* host. Mutants were then recombined into the *M. maripaludis* chromosome (92). Later, *in vivo* transposon mutagenesis in the acetoclastic methanogen, *M. acetivorans* C2A, was demonstrated. A modified insect *mariner*-family transposable element, *Himar1*, was used to generate random mutations. By identifying mutations that interfered with the use of methanol for methanogenesis, the function of the *hpt* gene, which encodes for hypoxanthine phosphoribosyl transferase, was identified (93). The designed transposable element, *Himar1*, can also function in distantly related species, such as *M. maripaludis* (93). A modified mini-*Himar1* element has also been used to identify genes required for formate-dependent growth in *M. maripaludis* (94). The mini-MAR367 element was then used to demonstrate the involvement of HrsM, a LysR-type transcriptional regulator, in the selenium-dependent gene expression of *M. maripaludis* (51). More recently, these transposon-mediated methods were used to identify the genes required for DNA uptake and transformation in *M. maripaludis* (71).

Separately, the function of proline biosynthesis genes in *M. acetivorans* and the function of tryptophan biosynthesis genes in *M. maripaludis* were determined using the Tn5 transposon inserted into the tryptophan operon, followed by transformation into *M. maripaludis* (95). Later, a Tn5 transposon derivative was used to conduct a comprehensive whole-genome analysis of gene function in *M. maripaludis* by generating libraries of transposon mutants (96). Although transposon-mediated mutation may be useful in studies requiring random mutagenesis (e.g., determining essential genes), the relatively understudied genome of many methanogens and the time-intensive nature of this method limits its application in many methanogen studies (43).

### Liposome-mediated mutagenesis

In contrast with random mutagenesis methods, liposome-mediated mutagenesis may be used for site-directed mutagenesis. Liposomes use a positively charged lipid to form a protective packet around a negatively charged DNA molecule. When mixed with spheroplasts, the positively charged lipid exterior binds to the negatively charged microbial cell membranes. The cell then imports the liposome, and the liposomal membrane is discarded. The released DNA can then be incorporated into the microbial genome through various mechanisms, such as recombination or integration. Liposomes facilitate the entry of DNA into cells and protect the DNA from degradation by extracellular nucleases, thereby enhancing the efficiency of DNA uptake. Additionally, liposomes can encapsulate a wide range of DNA sizes and types, including plasmids, linear DNA fragments, or oligonucleotides. However, the success of liposome-mediated transformation is highly dependent on the specific microorganism, liposome composition, and transformation conditions (12, 41, 97, 98).

The first reported liposome-based transformation method for *M. acetivorans* used a naturally occurring plasmid, pC2A. DOTAP (1,2-dioleoyl-3-trimethylammonium-propane), a cationic lipid, was mixed with Hepes buffer and the plasmid to form a DNA:liposome complex. Transformation was then achieved by mixing the complex with cells and incubating it at room temperature (41). A slightly modified procedure was used to transform *M. acetivorans* using a plasmid containing the gene for methyl-coenzyme M reductase (Mcr) from anaerobic methanotrophs by incubating at 37°C (99). Liposome-mediated transformation has also been used in *M. acetivorans* to examine proline biosynthesis genes (39), and to express *mekB*, a gene encoding a broad-specificity esterase from *Pseudomonas veronii* (100). A method for high-efficiency liposome-mediated transformation has been developed for *M. acetivorans* C2A and *M. barkeri* Fusaro (101). Furthermore, a liposome-mediated transformation protocol for *Methanosarcina mazei* strain Gӧ1 has been developed (97), which has been used in subsequent studies to insert a shuttle vector into *M. mazei* (62), and to develop the first functional genetic system for a virus of methanogens, MetSV (102).

Liposome-mediated transformation does not introduce any viral vectors, is relatively efficient, and has low cytotoxicity. However, the introduced DNA may not integrate into the chromosomal DNA, which may result in transient gene expression over multiple cell divisions. Long DNA strands are ineffectively introduced into the cell, thereby constraining the quantity of genetic material transferable in each transformation. Liposome-mediated transformation methods are also labor-intensive and can require the tuning of method parameters, such as liposome-to-DNA ratio, incubation time, and temperature (103).

## MARKERLESS GENETIC EXCHANGE

To date, two markerless genetic exchange techniques, homologous recombination and CRISPR- (Clustered Regularly Interspaced Short Palindromic Repeats-) Cas (CRISPR-associated) systems, have been applied to methanogens.

### Homologous recombination

Homologous recombination is a natural cellular process that exchanges the genetic material between two DNA molecules that have similar (i.e., homologous) sequences, and is used for genetic repair and recombination. Although homologous recombination allows for precise targeted gene editing, in some cases, genomic integration can require a long homology arm, which increases the genetic construct length and decreases the likelihood that the cell will successfully uptake the construct (77, 104, 105).

Markerless genetic exchange with homologous recombination typically uses both selection and counterselection markers (see “Selection and counterselection markers”). Selection markers are used to select cells that have integrated the desired DNA sequences into their genome. Counterselection markers, such as toxic genes, can then be employed to eliminate cells that retain the original, unmodified DNA, ensuring that only cells with the desired genetic modification are selected and propagated. For example, selection based on histidine auxotrophy/prototrophy using the *hisA* gene was demonstrated in *M. voltae* (106). *M. maripaludis* can be counterselected by targeting its growth sensitivity to 8-azahypoxanthine (via hypoxanthine phosphoribosyltransferase, *hpt* gene) and 6-azauracil (via uracil phosphoribosyltransferase, *upt* gene) (107).

A markerless genetic exchange technique was developed for *M. acetivorans* C2A by utilizing the discovery that mutants lacking the *hpt* gene are approximately 35 times more resistant to 8-aza-2,6-diaminopurine (8ADP) than the wild type (38). A desired mutation was cloned into a non-replicating plasmid, pMP44, which contained the selectable marker for puromycin resistance, *pac*, and the counter-selectable marker for 8ADP sensitivity, *hpt*. The plasmid was used to transform *M. acetivorans*, and resulting merodiploids were sensitive to 8ADP because of the *hpt* gene on pMP44. Nonselective growth conditions promoted plasmid excision in a second recombination event, which resulted in either the wild type or a recombinant that could be selected using 8ADP resistance. In this second recombination event, the plasmid backbone was removed from the chromosome, thereby producing a mutant that only differed from the wild type by the presence of the desired mutation (38). This markerless mutagenesis method has also been used to probe the role of three adjacent genes related to alanine metabolism in *M. maripaludis*, which has a unique ability among archaea to utilize both l- and d-alanine as a nitrogen source (107). Later, this method was demonstrated in *M. mazei* strain Gӧ1 and used to delete a gene encoding for a regulatory non-coding RNA (35).

### CRISPR/Cas-mediated genome editing

Prokaryotic CRISPR/Cas systems naturally occur as defense mechanisms against DNA viruses, but have been recently adapted for use in markerless genetic exchange techniques for microorganisms, including methanogens (43, 75, 80, 81, 105, 108–110). Although CRISPR/Cas systems are nearly ubiquitous within Archaea (unlike in Bacteria), only two model methanogens currently have well-developed CRISPR/Cas toolboxes: *M. acetivorans* and *M. maripaludis* (Table 3) (68, 75, 80, 81, 105, 111).

**TABLE 3:** CRISPR/Cas toolboxes for gene editing in methanogens

Methanogen	CRISPR/Cas system	Reference
*Methanococcus maripaludis*	CRISPR-Cas9	(68, 105)
	CRISPR/Cas12a	(75)
*Methanosarcina acetivorans*	CRISPR-Cas9	(111)
	CRISPRi-dCas9	(80)
	CRISPR/Cas12a	(81)

CRISPR/Cas systems can induce double-strand DNA breaks at specific genomic sites. Repair mechanisms, such as homology-directed repair (HDR), may then be used to introduce genetic modifications, thereby deleting, inserting, or replacing specific DNA sequences without relying on selectable markers (112). CRISPR/Cas systems can be divided into Class 1 systems, which are characterized by multi-subunit complexes and complex mechanisms, and Class 2 systems, which are simpler and feature single proteins for target recognition and cleavage. Within Class 2 systems, the Cas9 protein is used for its versatility in genome editing, while Cpf1 (Cas12) offers an alternative with distinct target recognition and cleavage properties.

CRISPR-Cas9 systems have been developed for both *M. acetivorans* and *M. maripaludis* (68, 105, 111). Methanogen CRISPR-Cas9 genome editing was first successfully demonstrated in *M. acetivorans*, which reduced the time required for mutant construction and simplified double mutant creation (111). Additionally, by co-expressing the non-homologous end joining (NHEJ) machinery from another archaeon, *Methanocella paludicola*, efficient genome editing was achieved without the need for a repair template, thereby offering a versatile genetic system for gene manipulation and functional studies in methanogenic archaea (111). Later, a Cas9-based system was developed in *M. maripaludis* to enable efficient and precise genome editing, including gene deletion, large DNA fragment removal, and single-nucleotide mutagenesis (105).

CRISPR/Cas systems may also be used to alter gene expression in other ways, which have largely been unexplored in methanogens. Currently, *M. acetivorans* is the only methanogen with a reported CRISPRi-dCas9 (CRISPR interference-dead Cas9) system (80), which is a variant of the CRISPR-Cas9 genome editing technology that is adapted for gene inhibition, rather than gene editing or modification. A CRISPRi-dCas9 system uses a modified Cas9 protein (dCas9) that does not cause genetic modifications but, instead, binds to a target gene’s DNA, preventing gene transcription without permanent genetic code alteration (80). Other CRISPR-based tools have yet to be demonstrated in methanogens, including CRISPR activation (CRISPRa) and CRISPR knockout (CRISPR KO).

In contrast to Cas9-based systems, CRISPR/Cas12 systems have the unique ability to process the CRISPR array within the same protein complex where it resides, which means that Cas12 can convert the spacers within the CRISPR array into mature crRNAs without the need for separate, externally synthesized sgRNAs. CRISPR/Cas12 toolboxes for gene editing have recently been developed for both *M. acetivorans* and *M. maripaludis* (75, 81). Bao et al. (75) described the first Cas12 toolbox for methanogens using Cas12a (LbCas12a) and endogenous HDR machinery in *M. maripaludis*, thereby enabling highly efficient gene deletions (up to 95% success rate) despite the microbe’s hyperpolyploidy. The toolbox was used to demonstrate a large gene deletion and provided a reliable and swift method for *M. maripaludis* genome editing, which is essential for metabolic engineering and biotechnological applications. More recently, Zhu et al. (81) described a CRISPR/Cas12 system for *M. acetivorans*, which allowed for the deletion of large genomic segments, efficient heterologous gene insertions, and the facilitation of multiplex genome editing. When used in conjunction with the Cas9-based system, it further accelerated targeted genome editing, offering a valuable tool for genetic engineering in Methanosarcinales species (81). In the future, CRISPR-based tools may be used to engineer microbial CO_2_-metabolizing chasses, such as methanogens, to produce fuels and valuable chemicals (113).

HDR and NHEJ represent two distinct mechanisms employed by CRISPR-based tools for gene manipulation. HDR relies on a donor template with homologous sequences to precisely insert or replace genes, while NHEJ often results in small insertions or deletions at target sites, making it suitable for gene disruption or knockout. Some host cells may already express genes for these mechanisms; however, constructs for genetic manipulation may express NHEJ machinery along with CRISPR tools to facilitate insertions or deletions without the use of homology scaffold for HDR after DNA double-strand break (111, 112).

New archaeal CRISPR-Cas systems have been discovered, including an archaeal Cas9, which could act as an alternative for the original bacterial Cas9 or as a new Cas system altogether (114). Indeed, many archaeal genetic engineering challenges may be overcome by using native CRISPR systems and genetic selection methods specific to Archaea. For example, bacterial Cas9 variants may not operate at the extreme temperatures or high salinity conditions required by some methanogens but a native CRISPR system will consist of enzymes already adapted to the extreme conditions (115). In combination with CRISPR tools, synthetic biology components can help elucidate metabolisms, create genetic circuits, and develop Archaea into well-developed model organisms. CRISPRi and CRISPRa tools are useful for investigating archaeal metabolisms, and components (e.g., inducible promoters, transcription factors, and riboswitches) provide an additional layer of control for genetic circuits and metabolic processes (116). Regulatory elements can also be used in cellular and cell-free synthetic circuits for applications, such as biosynthesis or biosensing (117, 118). Genetic tools developed for methanogens make these archaea promising genetic engineering chasses for genetic studies and biotechnological development. Furthermore, the emergence of transcriptional and post-transcriptional tools has great potential to expand the biotechnology applications for methanogens.

## CONTROLLING GENE EXPRESSION

Gene expression can be controlled by constitutive promoters, which control the level of consistent gene expression, and/or inducible promoters, which control gene expression in response to specific external conditions. Archaeal promoters are less well-characterized than bacterial promoters but some distinct differences have been identified. Bacterial promoters comprise −10 and −35 regions that are recognized by the sigma factor of RNA polymerase, which initiates transcription. In contrast, archaeal promoters exhibit transcription factor B recognition element and the TATA box, which interact with transcription factors to initiate transcription (119).

Constitutive promoters are used to drive the expression of desired genes or reporter systems in genetic constructs. Constitutive promoters can be used to drive the expression of RNA interference constructs or guide the expression of CRISPR/Cas9 components for gene silencing or knockout. Additionally, constitutive promoters may be used for ensuring reliable gene expression during metabolic engineering or high-throughput screening, or even increasing expression of genes related to bioproduction under industrial applications. For applications in *M. maripaludis*, most studies have used the constitutive promoter P*hmvA*, which is derived from the histone promoter in *M. voltae* (63, 120). Recently, a large library of 81 constitutive promoters and 42 ribosomal binding site sequences for *M. maripaludis* was described, expanding the available genetic tools (68).

Lie and Leigh (121) demonstrated that alanine induces an intermediate regulatory response in nitrogen fixation (*nif*) and glutamine synthetase (*glnA*) gene expression in *M. maripaludis*. A novel repressor protein, NrpR, that regulates the transcription of *nif* in *M. maripaludis* was subsequently identified (42, 122). Thus, expression of *nif* could be controlled by varying nitrogen sources among ammonia, alanine, and N_2_ (122). Temperature and the transcriptional activator, MMP1718, also affect archaellin-encoding *flaB2* gene expression in *M. maripaludis* (123, 124). An inorganic phosphate-dependent promoter for *M. maripaludis* was developed based on the promoter for the *pst* operon, which encodes for the ATP-binding cassette transporter (65).

Inducible promoters for *Methanosarcina* spp. have also been developed, including a bacterial tetracycline-regulated promoter system that prevents transcription in the absence of tetracycline within *M. barkeri* (45). An inducible promoter system for *M. mazei* was developed that activated in the presence of trimethylamine (40). However, further research is needed to develop new inducible promoters that utilize different pathways and/or affect other methanogens.

## CONCLUSION

Although there have been many recent advances in archaeal genetic engineering, new tools for methanogens are still needed. Extremophilic methanogens, in particular, have significant biotechnology potential but lack many genetic tools. Additionally, the development of CRISPR-based techniques, such as CRISPR inhibition, activation, or knockout, holds promise for advancing the precision of genetic manipulations in methanogens. Furthermore, the development of transcriptional and post-transcriptional tools may help expand the biotechnological applications of methanogens. Future research should explore post-translational modifications and harness multi-omics data to improve methanogen performance and diversify their applications. The exploration of emerging research avenues, such as the manipulation of post-translational modifications and the integration of multi-omics data, may lead to breakthroughs in improving methanogen performance and expanding their applications. Such endeavors necessitate interdisciplinary collaboration spanning microbiology, molecular biology, bioinformatics, environmental engineering, and environmental science. By fostering collaborative efforts across diverse fields, the genetic engineering of methanogens is poised to evolve into practical and sustainable solutions to contemporary challenges.
